# Fanless, porous graphene-copper composite heat sink for micro devices

**DOI:** 10.1038/s41598-021-97165-y

**Published:** 2021-09-02

**Authors:** Hokyun Rho, Yea Sol Jang, Hyojung Bae, An-Na Cha, Sang Hyun Lee, Jun-Seok Ha

**Affiliations:** 1 Chonnam National University, Gwangju, 61186 South Korea; 2 Korea Electronics Technology Institute, Seongnam-si, Gyeonggi-do 13509 South Korea

**Keywords:** Electronic devices, Graphene, Structural properties, Two-dimensional materials

## Abstract

Thermal management in devices directly affects their performance, but it is difficult to apply conventional cooling methods such as the use of cooling liquids or fans to micro devices owing to the small size of micro devices. In this study, we attempted to solve this problem by employing a heat sink fabricated using copper with porous structures consisting of single-layer graphene on the surface and graphene oxide inside the pores. The porous copper/single-layer graphene/graphene oxide composite (p-Cu/G/rGO) had a porosity of approximately 35%, and the measured pore size was approximately 10 to 100 µm. The internal GO was reduced at a temperature of 1000 °C. On observing the heat distribution in the structure using a thermal imaging camera, we could observe that the p-Cu/G/rGO was conducting heat faster than the p-Cu, which was consistent with the simulation. Furthermore, the thermal resistance of p-Cu/G/rGO was lower than those of the p-Cu and pure Cu. When the p-Cu/G/rGO was fabricated into a heat sink to mount the light emitting diode (LED) chip, the measured temperature of the LED was 31.04 °C, which was less than the temperature of the pure Cu of 40.8 °C. After a week of being subjected to high power (1000 mA), the light intensity of p-Cu/G/rGO decreased to 95.24%. However, the pure Cu decreased significantly to 66.04%. The results of this study are expected to be applied to micro devices for their effective thermal management.

## Introduction

The thermal management of micro devices has become increasingly important as the internal density of their circuits has increased^1–3^. As the aggregation of the internal circuits in electronic devices has increased, the amount of current per unit area has increased significantly^4–6^. The heat generated by an electronic device shortens the lifetime of that device or causes the occurrence of a failure^7–10^. According to the Arrhenius equation, the lifespan of an electronic device is cut in half when its temperature increases by 10 °C^11,12^. Therefore, it is necessary to develop heat sinks that can immediately release the heat generated by electronic devices. One of the properties required for effective thermal management with heat sink in electronic devices is heat dissipation properties. If the heat dissipation of heat sink is low, the temperature will rise due to continuous heat supply, and the device will be damaged^13–15^. Fans are used to improve the heat dissipation of heat sinks. However, it is difficult to attach fans to micro devices owing to the large volume, additional power requirement, and noise generation of fans. The development of a fanless heat sink is a challenge for future thermal management. A high heat dissipation is required in the case of a fanless heat sink, and a wide surface area and radiation rate are required for realizing a high heat dissipation^16–18^. Therefore, structures comprising a wide surface area, such as porous structures, are advantageous, and the use of materials having a high radiation rate is one solution to the aforementioned problem. Another well-known requirement of heat sinks is their high thermal conductivity^19–21^. Traditionally, primarily metals, which have a high heat conductivity, were used in heat sinks, but a significant amount of research was conducted to realize a higher heat conductivity by adding fillers to the metals^22–26^. Graphene, carbon nanotubes, fullerene, and diamond, which are carbon materials that have a high thermal conductivity, have been widely studied for such applications^26–28^. In particular, graphene has a very high thermal conductivity of 4000 W/mk. However, the range of improvements that can be realized in the characteristics of the actual heat dissipation material is small, because the ratio of the total density is low and the aspect ratio of the heat conductivity is large owing to its stratified structure^29–31^.

In this study, p-Cu/G/rGO was manufactured using copper powder, graphene oxide, and single-layer graphene surrounding the copper surface. The composite was identified as a porous structure having a porosity of approximately 35%. Single-layer graphene was synthesized on the surface of copper, and the GO inside was reduced at 1000 °C. The thermal conductivity of p-Cu/G/rGO was higher than that of p-Cu, and the temperature distribution analysis performed using a thermal imaging camera showed the same result as the simulation. The composite was fabricated into a heat sink that was used to mount the light emitting diode (LED), and the thermal management characteristics of the composite were analyzed.

## Result and discussion

**Figure 1:**
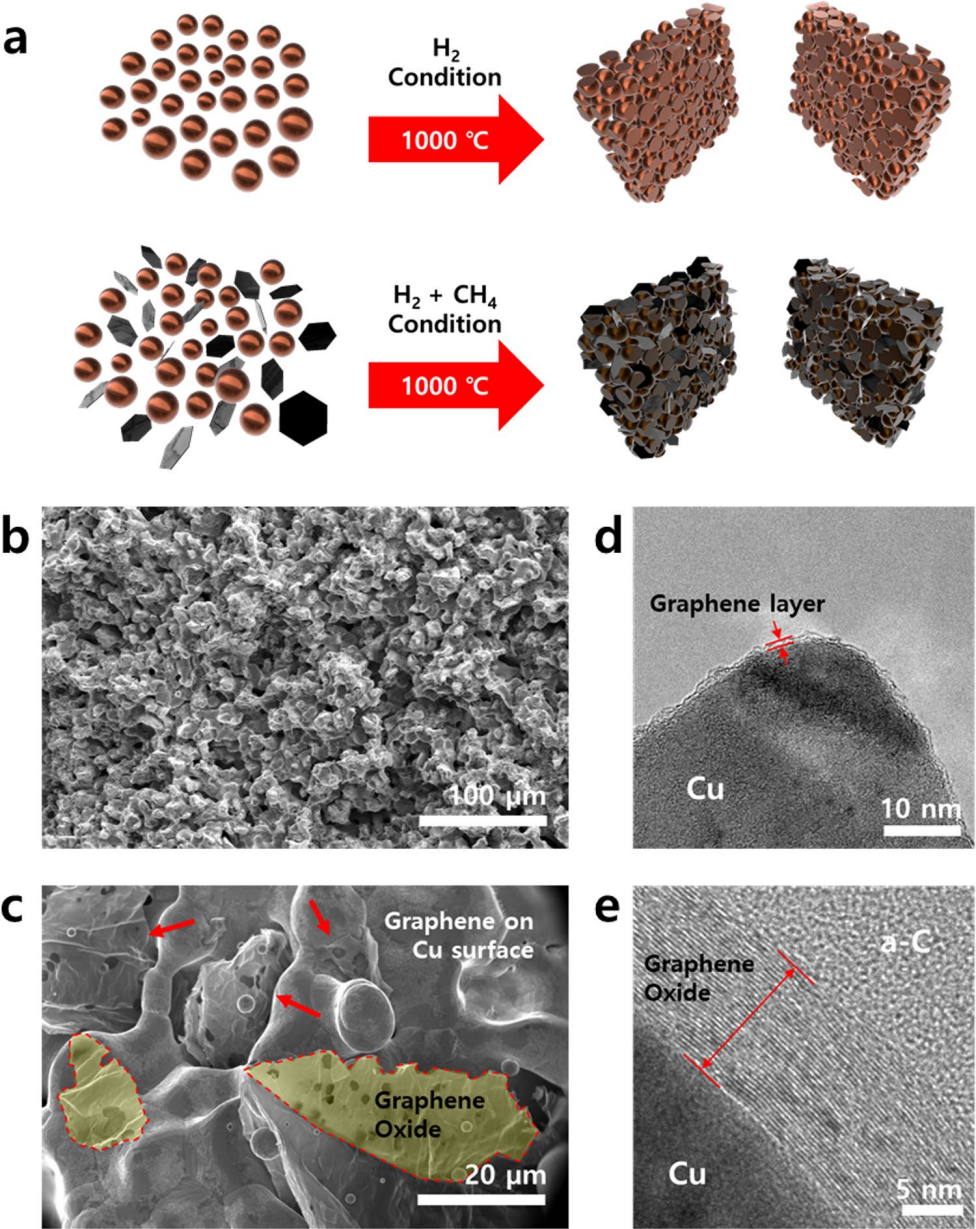
Schematic of a method for synthesizing p-Cu/G/rGO (**a**). SEM image of the internal structure of the p-Cu/G/rGO (**b**,**c**). TEM analysis of graphene and graphene oxide formed on a porous copper surface (**d**,**e**).

Copper powder of 5-μm particle size and graphene oxide were mixed and placed in a boat of 15 × 40 mm for synthesizing the graphene by thermal chemical vapor deposition. In order to confirm the structural characteristics, the surface of the composite was observed using scanning electron microscopy (SEM). The results are shown in Fig. 1. The observed p-Cu/G/rGO comprised a porous structure with graphene oxide inside its pores. Transmission electron microscopy (TEM) was used to observe the surface in greater detail. A single layer of graphene was formed on the surface of the copper, and it was also found that the graphene oxide was attached to the copper. This structure was created as follows. A 5-µm-sized copper powder was welded to the other copper powders around it, with the surface melted at a temperature of 1000 °C to form a porous structure. The surface melted at 1000 °C, below the melting point of copper, i.e., 1024 °C, owing to the lack of an outermost copper bond. The copper surface, which lacks atomic coupling, becomes unstable and melts at a temperature lower than its melting point^32,33^. This causes only the surface of the copper powder to be welded and a porous structure to form. At this time, the graphene oxide desorbs oxygen at the defect site owing to the temperature of 1000 °C and is reduced to graphene^34–36^. The reduced graphene oxides are bonded to a liquid copper surface. When methane is injected, the carbon atoms are then separated and dissolved in the porous copper at 1000 °C. In the cooling step, the carbon precipitates to grow into graphene on the surface of the copper and connects the reduced graphene oxide to result in a three-dimensionally connected graphene shape^37–39^.

**Figure 2:**
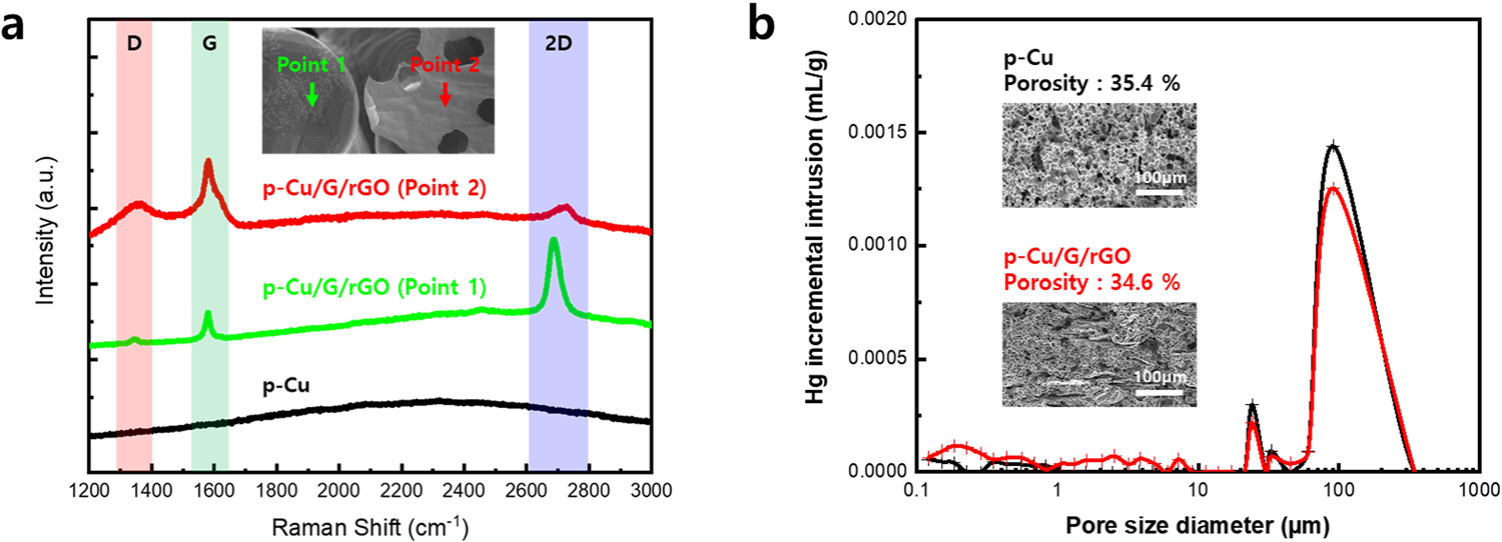
Raman spectroscopy of graphene and graphene oxide portions on copper surfaces (**a**). BET analysis for internal porosity and pore size confirmation (**b**).

The prepared p-Cu/G/rGO was analyzed using Raman spectroscopy and is shown in Fig. 2. The parts that appeared to be copper and those that appeared to be reduced graphene oxide were measured respectively, and the porous copper samples produced using only copper powder were also compared. In Raman spectroscopy, graphene is usually identified by peaks called D(~ 1350 cm^−1^), G(~ 1580 cm^−1^), and 2D(~ 2690 cm^−1^). The D-peak is caused by disordered oscillation in sp^2^ bonds (generally a defect), and the G peak arises from the stretching of the C–C bond in graphitic materials and is common to all sp^2^ carbon systems. The 2D-peak is caused by a second-order two-phonon process. In addition, the intensity comparison of the G-peak and 2D-peak provides information regarding the number of graphene layers^40–43^. In the part that appears to be copper, the ratio of the G and 2D peaks is approximately 1:3, and it seems that a single layer of graphene exists. In the part that appears to be graphene oxide, the intensity of the G-peak is very high as compared to that of the 2D peak, and thus, the graphene oxide is considered to be reduced. Compared to the single-layer graphene, the 2D peak position of the reduced graphene oxide is shifted owing to their structural differences. The peaks of the reduced graphene oxide may not perfectly match those of the single-layer graphene owing to the up-and-down interference caused by the multi-layer structure and irregular atomic behavior due to several defects. No peaks were observed for the p-Cu samples prepared using only copper powder. The porosity of the porous structure was measured via BET (Brunauer–Emmett–Teller) analysis. The porosity was approximately 34.6%, and pores of approximately 10 to 100 µm in size were measured. In order for convection to occur within the open pores, the pore diameter should be 10 times the air's mean free path (MFP)^44,45^. In other words, the MFP of the air is approximately 64 nm, and heat exchange is thus possible if the pore diameter is greater than 640 nm.

**Figure 3:**
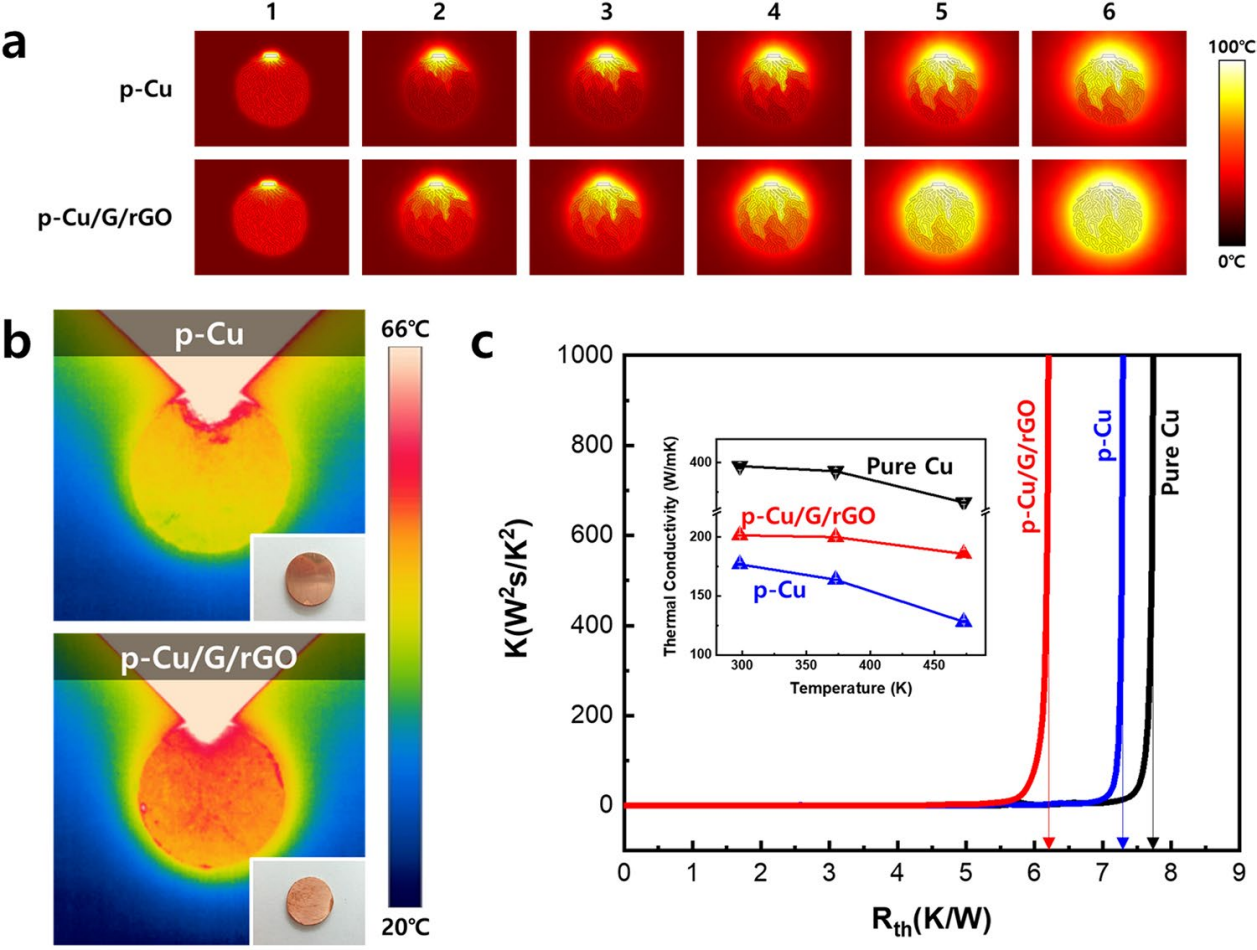
Heat transfer simulation results with and without graphene and graphene oxide in the structure (**a**). Thermal imaging camera analysis to verify thermal conductivity on real samples under the same conditions as the simulation (**b**). Analysis of thermal resistance (**c**) and thermal conductivity (inset) of composites.

A simulation was performed to confirm the heat dissipation ability of p-Cu/G/rGO, and is shown in Fig. 3a. The heating source was placed on top of the p-Cu and p-Cu/G/rGO samples, and the movement of the heat was observed by the same time step. Based on the simulation results, it can be confirmed that the heat transfer in the p-Cu/G/rGO sample is faster than that in the p-Cu sample. This result agrees with that obtained with a real sample (radius 10 mm × thickness 5 mm) in Fig. 3b and measured with a thermal imaging camera. Because of their porosity, p-Cu and p-Cu/G/rGO exhibited lower thermal conductivities than that of the pure Cu. The thermal conductivity of a material with a porosity of 35% is reduced to about 43% of that of a non-porous material (Supporting information S3)^46,47^.

This value is consistent with the measured value. The thermal conductivity of all the samples decreases as the measurement temperature increases, because of the increase in the thermal resistance due to the increased heat. However, in the case of p-Cu/G/rGO, the thermal conductivity decrease is small because graphene is coated onto the copper surface to prevent oxidation^48–50^. Furthermore, the increase in the thermal resistance due to the heat generation was confirmed using a thermal resistance tester, and is shown in Fig. 3c. Thermal resistance is the temperature difference across a structure when a unit of heat energy flows through it in unit time. The measured thermal resistances of P-Cu/G/rGO p-Cu, and pure Cu were 6.20, 7.30, and 7.74 K/W, respectively, when the incident thermal energy was 1000 W^2^∙s/K^2^. That is, for the same thermal energy, p-Cu/G/rGO had the lowest thermal resistance. Therefore, it can be observed that it has excellent heat dissipation properties. The reason it has a high heat dissipation property even though its thermal conductivity is lower than that of pure Cu is because of its convection and radiation effect. The heat dissipation property is not solely influenced by thermal conductivity but is a complex property that is affected by convection and radiation simultaneously. Irrespective of how quickly heat is transferred from inside the heat sink, if the ability to dissipate the heat outside the heat sink is low, the heat is accumulated in the heat sink and the temperature of the device increases. Therefore, if the heat conductivity is above a certain value, the factors that determine the performance of the heat sink are convection and thermal radiation. Convection and radiation are advantageous when the surface area is wide, and since the emissivity of graphene is approximately nine times greater than that of copper, p-Cu/G/rGO has excellent heat dissipation properties^51–53^.

**Figure 4:**
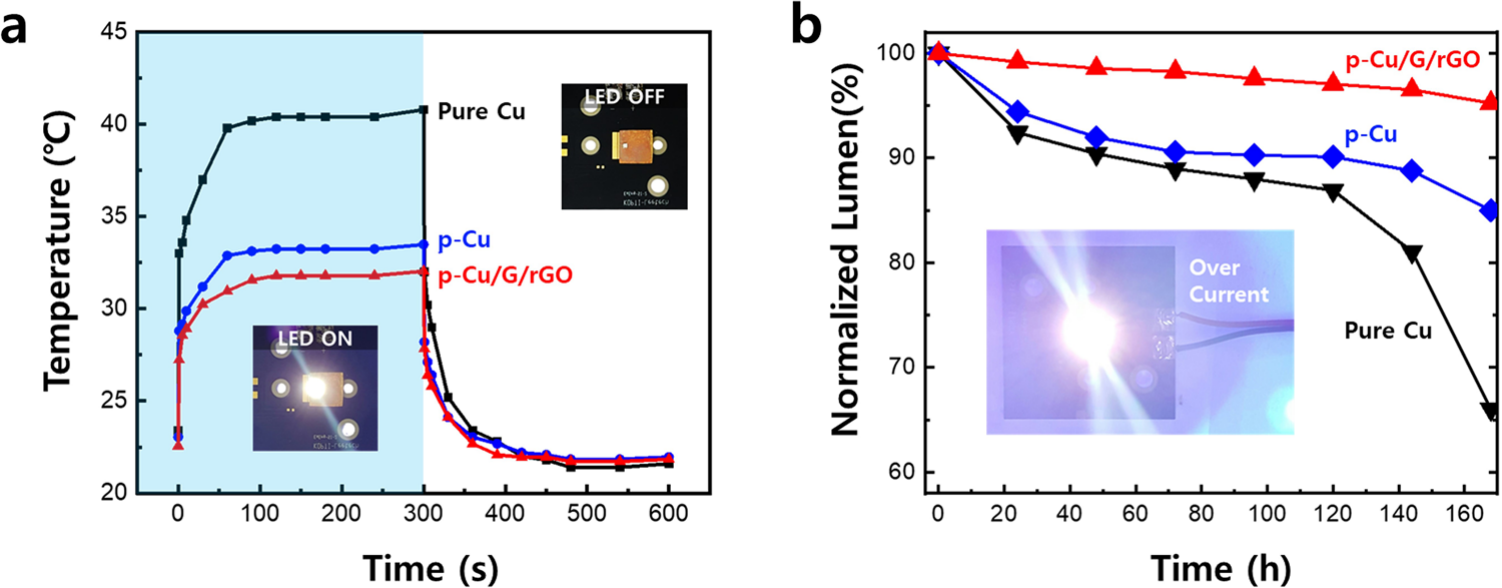
Graph of temperature change according to on/off of LED mounted on each heat sink (**a**). Graph of changes in brightness of the LEDs emitting under high power.

We used the composite to fabricate a heat sink of dimensions 10 × 10 × 1 mm^3^ and mounted the LED on the heat sink. In the temperature measurement method, 350 mA was applied for 5 min to generate heat, and the current was then cut off. After 5 min, the measured temperature of the LED chip was 31.04 °C, 33.48 °C, and 40.8 °C in the cases of p-Cu/G/rGO, p-Cu, and pure Cu heat sinks, respectively. This result is shown in Fig. 4a. The hot air delivered from the surface of the heat sink moves up owing to the density difference, and cold air is supplied from below to create natural convection. In such a case, porous structures have a large surface area where heat exchange can occur, which is advantageous for heat dissipation. The graphene on the porous surface makes the surface hydrophobic, thus encouraging heat exchange^54^. Therefore, the LEDs mounted on the p-Cu/G/rGO can exhibit the lowest temperature. Next, the stability of the LED chip was measured and shown in Fig. 4b. Because LEDs have a very long lifespan of approximately 50,000 h, experiments were conducted under high power of 1000 mA, which is three times the recommended driving voltage of the corresponding LED chip. The experiment was conducted over 1 week, and the brightness of the LEDs decreased to 95.24%, 84.99%, and 66.04% of the original value in the cases of the p-Cu/G/rGO, p-Cu, and pure Cu heat sinks, respectively. The reason for the sharp decrease in brightness is that the heat generated by the multi-quantum well of the LED is not emitted and accumulates, thus causing damage to the LED^55^. The p-Cu/G/rGO heat sink quickly dissipates the heat generated from the LED chip such that the heat does not accumulate at the LED, thereby resulting in a high stability.

## Conclusion

In this study, copper, graphene, and graphene oxide powders were used to produce composites with excellent heat dissipation properties. The produced p-Cu/G/rGO composite formed a porous structure, a single layer graphene was formed on the copper surface, and it was confirmed that reduced graphene oxide exists between the porous structures. The p-Cu/G/rGO was confirmed to have a good heat dissipation capability owing to its wide surface area and the presence of graphene, which was in agreement with the results of the simulation. The temperature of the LED chip was measured by fabricating a heat sink using the p-Cu/G/rGO and mounting the LED chip onto it. The LED mounted on the p-Cu/G/rGO heat sink exhibited the lowest temperature of 31.04 °C and maintained a brightness of 94.24% of the original value in the LED reliability test even under severe conditions. This p-Cu/G/rGO performance is expected to be incorporated into micro devices such as laptops, tablets, and cell phones for their heat management.

## Methods

### p-Cu/G/rGO manufacturing method

The copper powder having a particle size of 5 µm and the graphene oxide were mixed at a weight ratio of 50:1 and placed in an aluminum-oxide boat. The temperature was then increased to 1000 °C for 1 h in a hydrogen atmosphere. Methane was injected at a temperature of 1000 °C and naturally cooled to room temperature after 30 min. For comparison, p-Cu was prepared only with copper powder having a particle size of 5 µm and without the injection of methane and graphene oxide. High-purity (99.9%) Cu was purchased from Taewon Scientific Cooperation in Korea.

### Analysis of structural properties

The morphologies of the synthesized porous structures were characterized using an SEM system (Nova NanoSEM 450, FEI) and a 200-kV field-emission TEM system (JEM-2100F HR, JEOL). The graphene structure was confirmed using a Raman spectrophotometer (NRS-5100, JASCO). BET (AutoPore IV 9500, Micromeritics) was used to confirm the pore size and porosity.

### Analysis of thermal properties

The composite produced for the measurement of the thermal imaging camera (FLIR T-335, FLIR systems, spectral range 7.5–13 μm) was processed to a diameter of 20 mm and thickness of 5 mm. A heat source at 100 °C was contacted with the silver paste on top of the composite. To obtain precise thermal properties, i.e., by preventing the occurrence of surface scattering, the surface was treated using a commercial graphite spray (GB396352, Netzsch). The simulation was performed using the COMSOL Multiphysics software (ALTSOFT). The thermal diffusivity and specific heat were measured using the laser flash method (LFA-457, Netzsch) and a differential scanning calorimeter (DSC 8500, Perkin Elmer) at Energy Convergence Core Facility in Chonnam National University, respectively.

### Analysis of LED properties

The LED chip (size 1 × 1 mm^2^) used in this experiment has a vertical structure and driving current of 350 mA. The three composites (p-Cu/G/rGO, p-Cu, and pure Cu) were used to fabricate heat sinks for the LED chip. The LED chip was mounted onto each heat sink using a silver paste. Aluminum-based printed circuit boards were attached to the heat sinks. The thermal resistance of the LED device was measured using a thermal resistance tester (T3Ster, MicReD). A lifetime test was conducted on the LED under high power of 1000 mA and using an integrating sphere photometer (BTS256 LED Tester, Gigahertz Optik).

## Supplementary Information

Supplementary Information.
