# Supplementary material for: Fanless, porous graphene-copper composite heat sink for micro devices
Source: Sci Rep. 2021 Sep 2;11:17607. doi: 10.1038/s41598-021-97165-y (PMC8413455; doi:10.1038/s41598-021-97165-y)
Supplement: Supplementary file 1 — Supplementary Information. [file 41598_2021_97165_MOESM1_ESM.docx]

Supporting information

Fanless, Porous graphene-copper composite heat sink for micro devices

*Hokyun Rho,^a^ Yea Sol Jang,^b^ Hyojung Bae,^a^ An-Na Cha,^a^ Sang Hyun Lee^*,a^ and Jun-Seok Ha^*,a^*

*^a^* Chonnam National University, Gwangju 61186, South Korea

*^b^* Korea Electronics Technology Institute, Seongnam-si, Gyeonggi-do 13509, South of Korea

* corresponding author

**e-mail:** jsha@jnu.ac.kr, leeshyun@chonnam.ac.kr

**Keywords:** Thermal management, Graphene, Graphene oxide, Porous structure, Heat sink

**S1. SEM image of Cu powder**

**
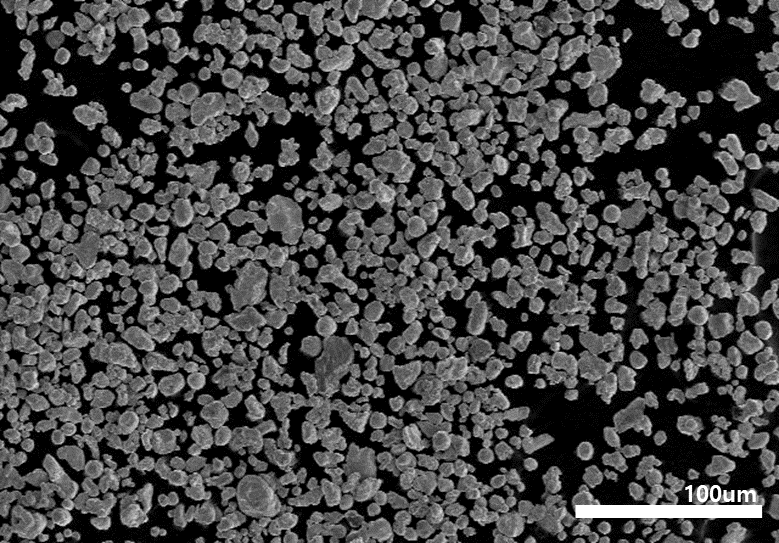
**

Figure S1. SEM image of Cu powder. Copper powder with an average size of 5 micrometers was used for the experiment.

**S2. p-CuGrGO with copper removed**

**
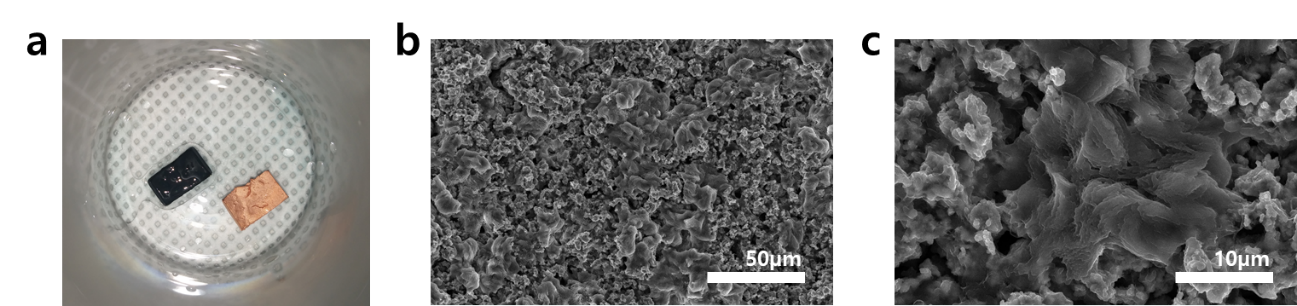
**

Figure S2. Picture of melting copper in p-CuGrGO using nitric acid(a). SEM image of an p-CuGrGO with copper removed(b,c).

**S3. The relationship between porosity and thermal conductivity.**


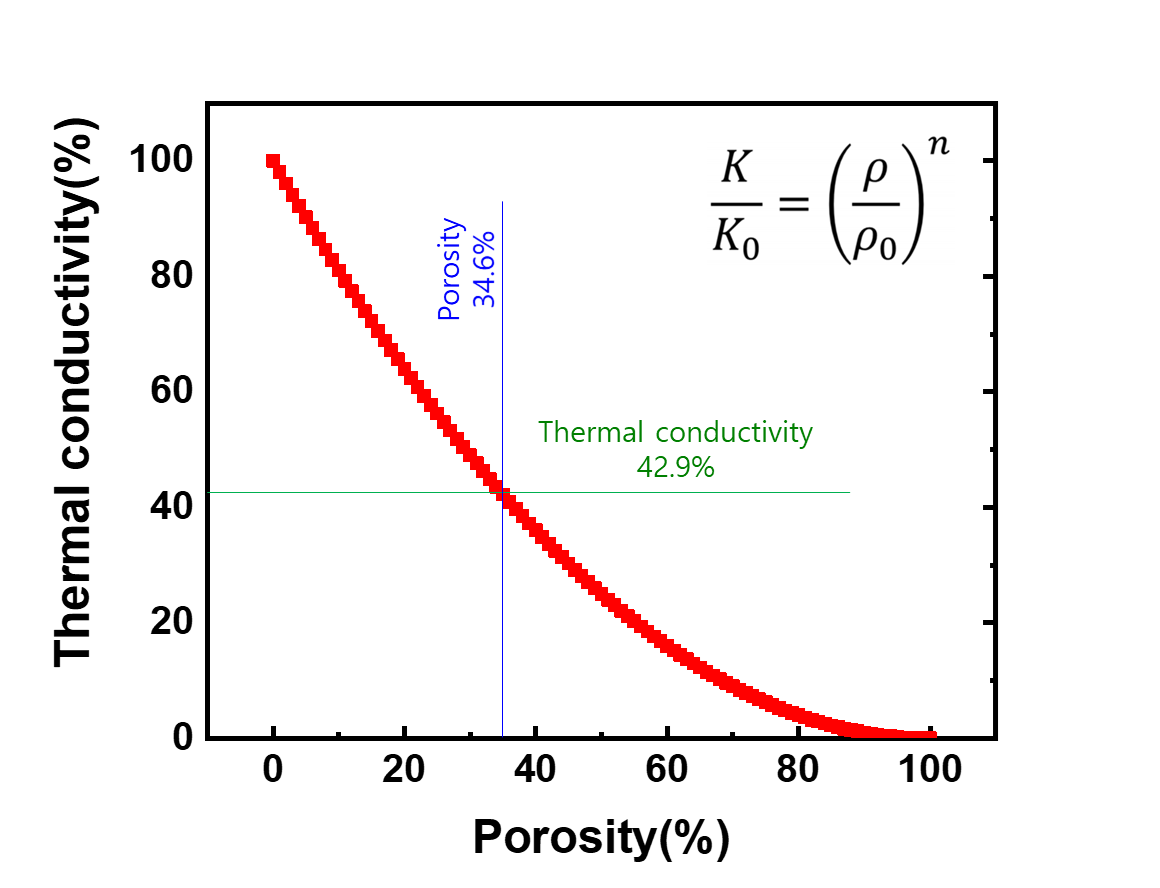


Figure S3. Graph of porosity and thermal conductivity.

As the porosity increases, the thermal conductivity decreases and the degree of decrease follows the following equation.

$$\frac{K}{K_{0}}=\left( \frac{\rho}{\rho_{0}} \right)^{n}$$

$K$ : thermal conductivity of the porous material

$K_{0}$ : thermal conductivity of the bulk material

$\rho$ : density of the porous material

$\rho_{0}$ : density of the bulk material

$n$ : exponent for thermal conductivity
